# ApoE isoforms differentially regulates cleavage and secretion of BDNF

**DOI:** 10.1186/s13041-017-0301-3

**Published:** 2017-06-01

**Authors:** Abhik Sen, Thomas J. Nelson, Daniel L. Alkon

**Affiliations:** Blanchette Rockefeller Neurosciences Institute, 8 Medical Center Drive, Morgantown, WV 26505 USA

**Keywords:** Alzheimer’s disease (AD), Apolipoprotein E (ApoE), Brain-derived neurotrophic factor (BDNF)

## Abstract

Apolipoprotein E4 (ApoE4) is a major genetic risk factor for sporadic or late onset Alzheimer’s disease (AD). Brain-derived neurotrophic factor (BDNF) is decreased by 3 to 4-fold in the brains of AD patients at autopsy. ApoE4 mice also have reduced BDNF levels. However, there have been no reports relating the different ApoE isoforms or AD to differential regulation of BDNF. Here we report that in the hippocampal regions of AD patients both prepro-BDNF and pro-BDNF expression showed a 40 and 60% decrease respectively compared to that expression in the hippocampi of age-matched control patients. We further report that ApoE isoforms differentially regulate maturation and secretion of BDNF from primary human astrocytes. After 24 h, ApoE3 treated astrocytes secreted 1.75- fold higher pro-BDNF than ApoE2-treated astrocytes, and ApoE2-treated astrocytes secreted 3-fold more mature-BDNF (m-BDNF) than ApoE3-treated astrocytes. In contrast, ApoE4-treated cells secreted negligible amounts of m-BDNF or pro-BDNF. ApoE2 increased the level of intracellular pre-pro BDNF by 19.04 ± 6.68%, while ApoE4 reduced the pre-pro BDNF by 21.61 ± 5.9% compared to untreated cells. Similar results were also seen in ApoE2, ApoE3 or ApoE4 treated cells at 4 h. Together, these results indicate that an ApoE2 or ApoE3 mediated positive regulation of BDNF may be protective while ApoE4 related defects in BDNF processing could lead to AD pathophysiology. These interactions of the ApoE isoforms with BDNF may help explain the increased risk of AD associated with the ApoE4 isoform.

## Introduction

The discovery of the apolipoprotein E (ApoE) 4 allele as a major risk factor for sporadic and late-onset familial AD has brought attention to the possible role of ApoE in neurodegenerative conditions. The human ApoE gene exists as three polymorphic alleles—ε2, ε3 and ε4—which have a worldwide frequency of 8.4, 77.9, and 13.7%, respectively [1]. The presence of the ApoE4 allele is associated with increased risk of cerebral amyloid angiopathy and age-related cognitive decline during normal aging [2]. The ApoE4 allele is also a risk factor for other neurodegenerative conditions, such as Parkinson’s and multiple sclerosis, and is associated with worse outcomes after ischemic or traumatic brain injury [3].

In the CNS, the ApoE protein is mainly produced by astrocytes and transports cholesterol to neurons via the low­density lipoprotein receptor (LDLR) family. ApoE is an essential component for axonal growth and synaptic formation and remodeling—events that are crucial for learning, memory formation, and neuronal repair. ApoE also regulates hippocampal neurogenesis by maintaining the neural progenitor cell pool in the adult dentate gyrus region of the hippocampus [4]. In AD and healthy aged controls, APOE ε4 gene dosage correlates inversely with dendritic spine density in the hippocampus [5]. Reduced synaptic transmission was observed in 1­month­old ApoE4­transgenic mice compared with ApoE3­transgenic mice, suggesting that ApoE4 may also contribute to functional deficits early in development, which may account for alterations in neuronal circuitry that result in cognitive disorders later in life [6]. ApoE4 not only increases Aβ deposition but also affects synaptic repair, plasticity and neural development [2, 7]. We have also shown that ApoE3 acts through PKCε to protect primary neurons against Aβ-induced cell death and induce synaptogenesis, whereas ApoE4 does not [8]. Furthermore, ApoE4 epigenetically suppresses BDNF mRNA expression by inducing nuclear translocation of histone deacetylases 4 and 6 (HDACs) in human neurons [9].

Brain-derived neurotrophic factor (BDNF) is a critical factor in synaptic repair and plasticity. Considerable evidence suggests that a decrease in BDNF is associated with AD. BDNF expression is greatly decreased in the hippocampus and temporal and frontal cortex of AD patients [10]. BDNF mRNA is decreased in parietal cortex and hippocampus by 3 to 4-fold in AD autopsy samples [11]; both precursor and mature BDNF are dramatically decreased in preclinical stages of AD, and correlate with behavioral scores [12]. Although evidence for BDNF polymorphisms in AD is still inconclusive, synaptic loss is the single most important correlate of AD. BDNF protein is synthesized as pre-proBDNF and cleaved into a proBDNF protein. ProBDNF is either cleaved intracellularly or extracellularly to mature BDNF (mBDNF). Pro-BDNF and mBDNF function differently although it has not been previously known whether ApoE isoforms differentially regulate the cleavage of pro-BDNF.

Astrocytes are a well characterized source for biologically active molecules including BDNF, both in culture and in vivo. Astrocytes are known to produce BDNF in vivo following injury [13]. In culture, they increase BDNF synthesis and release in response to glutamate metabotropic stimulation. Numerous glutamate signaling proteins are downregulated in ApoE4 mice [14]. Since ApoE in the brain is mainly produced by the astrocytes we investigated whether the ApoE isoforms have an effect on the maturation and secretion of BDNF from astrocytes.

## Methods

### Materials

Cell culture media were obtained from Sciencell, CA, USA. Recombinant human ApoE2 (rh-ApoE2), ApoE3 (rh-ApoE3), ApoE4 (rh-ApoE3), and other reagents were purchased from Sigma-Aldrich (St. Louis, MO, USA). Primary antibodies against BDNF and β-actin were obtained from Santa Cruz Biotechnology, Inc. (Santa Cruz, CA, USA); all secondary antibodies were purchased from Jackson ImmunoResearch Laboratories, Inc. (Bar Harbor, ME, USA).

### Culture of primary human astrocytes

Human primary astrocytes (ScienCell Research Laboratories, Carlsbad, CA, USA) were plated on poly-L-lysine coated plates and were maintained in astrocyte medium (ScienCell Research Laboratories, Carlsbad, CA, USA) supplemented with the astrocyte growth supplement (AGS, ScienCell Research Laboratories, Carlsbad, CA, USA) and 2% fetal bovine serum (FBS). For maintenance of astrocytes, the media was changed every 3 days. Before treatment of the cells, the media was replaced with serum and growth factor free DMEM/F12 media (Invitrogen, Carlsbad, CA). Cells were treated with cholesterol, or ApoE2/ApoE3/ApoE4 + cholesterol for 4 and 24 h. ApoE (20 nM) and cholesterol (100 μM) were added separately to the cultures. Cholesterol was dissolved in ethanol.

### Collection of cell secreted media

Media from the treated cells was collected using a pipette. The collected media was concentrated 10-fold using a 3-kDa molecular-cut filter (Amicon Ultra-4, Millipore, Tullagreen, Ireland).

### Human brain tissue

Fresh frozen human brain tissue was obtained from the Harvard Brain Tissue Resource Center, McLean Hospital, Boston, MA which is the part of NIH NeuroBioBank (IRB: Partners Human Research Committee; Title: Brain Tissue Repository for Research on Neurological and Psychiatric Disorders; IRB No: 2015P002028) (Table 1). Approval for the study was obtained from tissue access committee of NIH NeuroBioBank (https://neurobiobank.nih.gov/documents/). Informed consent was obtained from all patients or legal representatives. The pathological diagnosis of AD was conducted according to the Consortium to Establish a Registry for Alzheimer’s disease (CERAD). The study was carried out in accordance with the Code of Ethics of the World Medical Association (Declaration of Helsinki) for experiments involving humans (http://www.share-project.org/fileadmin/pdf_documentation/Declaration_of_Helsinki.pdf).

**Table 1:** Patient ID, Braak stage and age of human brain tissue

Patient ID	Gender	Age	Average age ± SD	Autopsy diagnosis
AN02930	M	80	77.5 ± 12.34	AD Braak 3
AN14554	F	61		AD Braak 6
AN17726	M	72		AD Braak 2
AN06468	M	98		AD Braak 4
AN16195	F	73		AD Braak 5
AN02773	F	81		AD Braak 5
AN00704	F	82	77.4 ± 5.86	Control
AN00316	F	75		Control
AN17896	M	69		Control
AN12667	M	86		Control
AN08396	M	76		Control
AN15515	M	73		Control
AN10329	F	81		Control

### Cell and tissue protein preparation

Cells and tissue were harvested in homogenizing buffer, containing 10 mM Tris-Cl (pH 7.4), 1 mM PMSF (phenylmethylsulfonyl fluoride), 1 mM EGTA, 1 mM EDTA, 50 mM NaF, 20 μM leupeptin and 1% NP-40 and were lysed by sonication. Protein concentration was measured using the Coomassie Plus (Bradford) Protein Assay kit (Pierce, Rockford, IL, USA).

### Immunoblot

Following quantification, 40 μg of protein from each sample was subjected to SDS-PAGE analysis in 4–20% gradient Tris-Glycine gel (Invitrogen, Carlsbad, CA, USA). The separated protein was then transferred to a nitrocellulose membrane. The membrane was blocked with BSA at room temperature for 15 min and was incubated with primary antibody overnight at 4 °C. After incubation, it was washed 3× with TBS-T (Tris-buffered saline-Tween 20) and further incubated with alkaline phosphatase conjugated secondary antibody (Jackson Immunoresearch Laboratories) at 1:10,000 dilution for 45 min. The membrane was finally washed 3× with TBS-T and developed using the 1-step NBT-BCIP substrate (Pierce, Rockford, IL, USA). The blot was imaged in the ImageQuant RT-ECL (GE Life Sciences, Piscataway, NJ, USA) and densitometric quantification was performed using IMAL software. For quantifying expression of a protein, densitometric values for the protein of interest were normalized against β-actin (loading control).

### Statistical analysis

All experiments were performed at least three times. Data are represented as mean ± SEM. Data containing more than two groups was analyzed using one-way ANOVA with post-hoc Newman-Keuls multiple comparison test. Data with two groups were tested for significance with paired two-tailed *t* test comparisons using GraphPad Prism 6.1 software (La Jolla, CA, USA). *P* values <0.05 were considered statistically significant.

## Results

### BDNF levels are lower in autopsy-confirmed human AD hippocampus

**Fig. 1:**
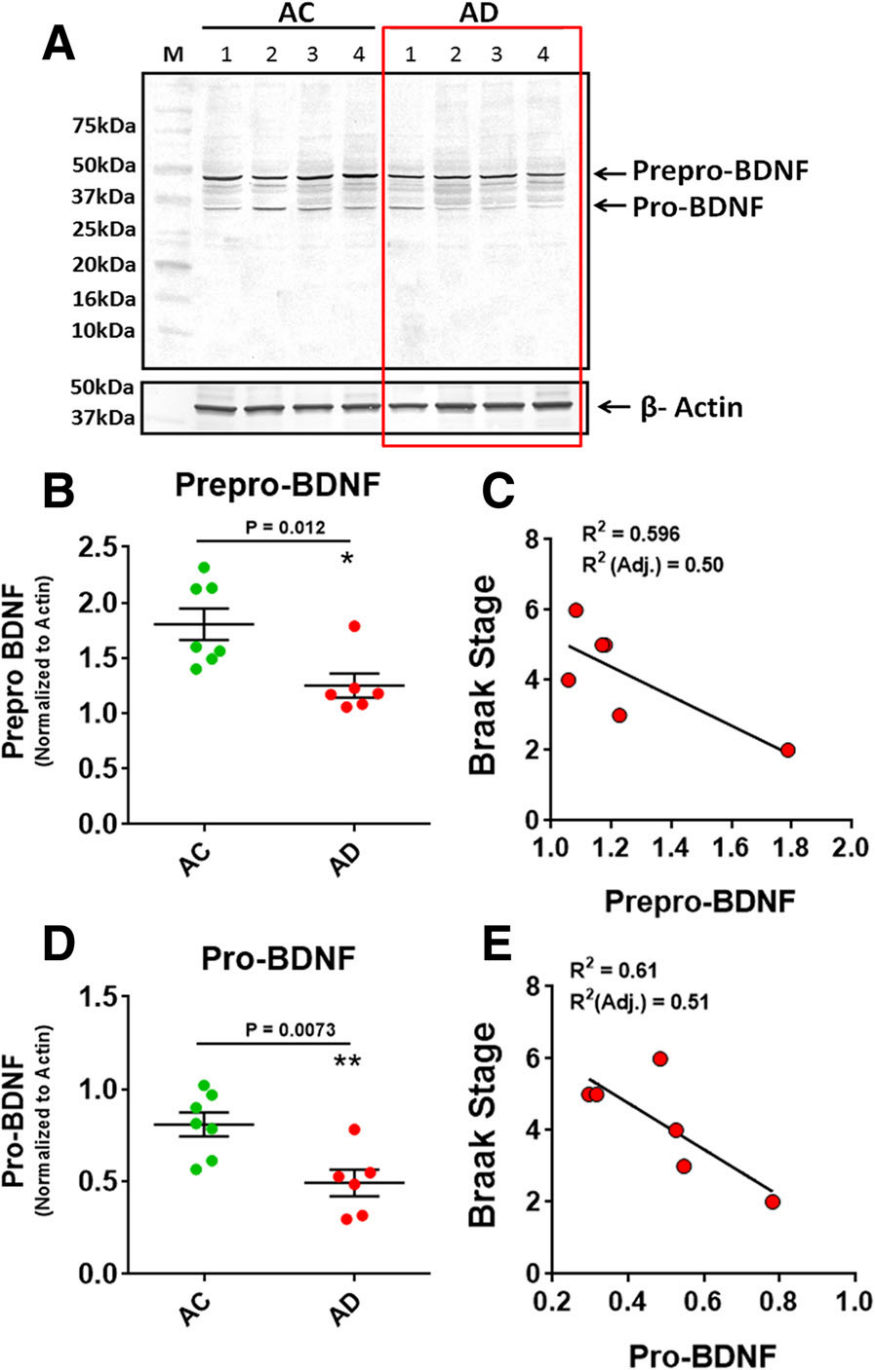
Human Alzheimer’s disease hippocampus has lower BDNF levels. **a** Immunoblot showing BDNF expression in the hippocampus of four AD and four age matched controls (AC). In AD hippocampus the prepro-BDNF levels were significantly lower than AC hippocampus (AC: 1.8 ± 0.14, *n* = 7; AD: 1.25 ± 0.11, *n* = 6. *P* = 0.012) **b** and negatively correlated with the Braak stage **c**. Pro-BDNF expression were also lower in AD hippocampus (AC: 0.81 ± 0.07, *n* = 7; AD: 0.49 ± 0.073, *n* = 6. *P* = 0.0073) **d**, **e**. Data are represented as mean ± SE (Student’s *t*-test, *P < 0.05 and **P < 0.005)

To determine if BDNF levels are affected in AD brain, we measured the BDNF levels from autopsy-confirmed human AD hippocampus (Average age = 77.5 ± 12.34 (SD); *n* = 6) and aged matched control (AC) human hippocampus (Average age = 77.4 ± 5.86 (SD); *n* = 7) using immunoblot. In AD hippocampus both the prepro-BDNF and pro-BDNF levels were significantly lower than AC hippocampus (Fig. 1b, d). No m-BDNF was detected in these samples. Both prepro-BDNF and pro-BDNF levels showed a negative correlation (not significant) with the Braak stage among AD cases (Fig. 1c, e).

### ApoE isoforms differentially regulate the secretion of mature BDNF from human astrocytes

To evaluate the effect of ApoE2, ApoE3 and ApoE4, primary human hippocampal astrocytes were grown in serum free media and treated with cholesterol (100 μM), ApoE2 (20 nM) + cholesterol, ApoE3 (20 nM) + cholesterol and ApoE4 (20 nM) + cholesterol for 4 and 24 h. After the treatment the media was collected and BDNF levels were measured by immunoblot.

**Fig. 2:**
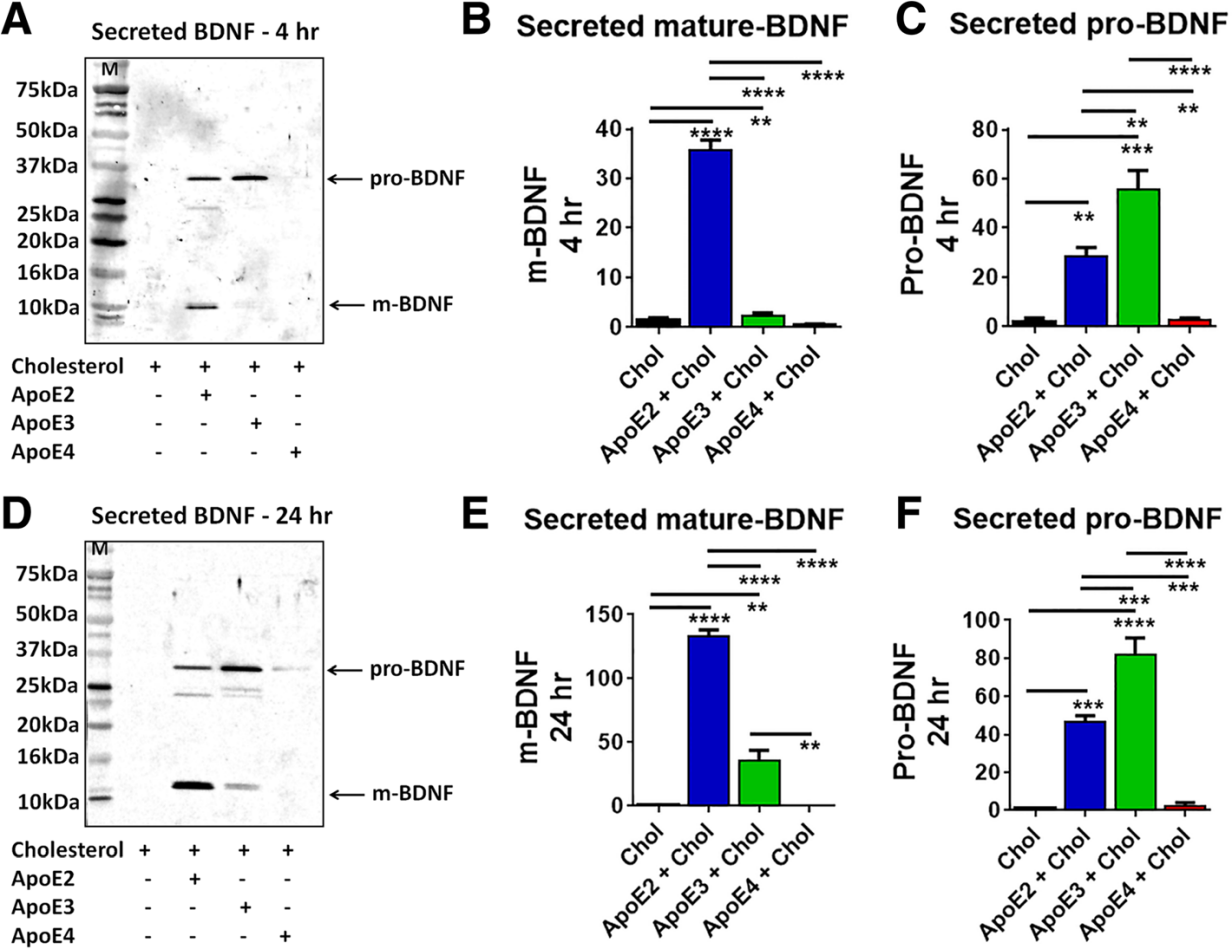
ApoE isoforms differentially regulate the secretion of mature-BDNF. Primary human astrocytes were treated with cholesterol (Chol; 100 μM), with or without ApoE2 (20 nM) or ApoE3 (20 nM) or ApoE4 (20 nM) for 4 and 24 h. **a** and **d** Immunoblot showing expression levels of BDNF in the astrocyte-secreted media after 4 and 24 h respectively. ‘M’ indicates molecular weight marker. **b** and **e** ApoE2 + cholesterol induce the secretion of m-BDNF. **c** and **f** Expression levels of secreted pro-BDNF in different ApoE isoforms treated cells. Data are represented as mean ± SE of three independent experiments. (*P < 0.05, **P < 0.005 ***P < 0.0005 and ****P < 0.00005; ANOVA, post hoc Newman-Keuls multiple comparison)

At 4 h the amount of secreted m-BDNF (14 kDa) was increased by 22.5-times in ApoE2 + cholesterol treated cells and 1.52-times in ApoE3 + cholesterol treated cells compared to only cholesterol treated cells. (ANOVA: F _(3,8)_ = 269.3; *P* < 0.0001) (Fig. 2a, b). After 24 h, secreted m-BDNF was increased by 143.5-fold in ApoE2 + cholesterol treated cells and 38.4 -fold in ApoE3 + cholesterol treated cells compared to only cholesterol treated cells (ANOVA: F _(3,8)_ = 151.3; *P* < 0.0001) (Fig. 2d, e). ApoE4 + cholesterol treated cells showed no change in secreted m-BDNF at 4 and 24 h.

Secreted pro-BDNF level was increased by 13.8- fold in ApoE2 + cholesterol treated cells and 30 -fold in ApoE3 + cholesterol treated cells compared to cholesterol-only treated cells (ANOVA: F _(3,8)_ = 32.0; *P* < 0.0001) after 4 h (Fig. 2a, c). At 24 h, secreted pro-BDNF levels increased by 48.7-fold in ApoE2 + cholesterol treated cells and by 85.4-fold in ApoE3 + cholesterol treated cells compared to cholesterol-only treated cells (ANOVA: F _(3,8)_ = 62.7; *P* < 0.0001) (Fig. 2d, f). ApoE4 + cholesterol treated cells showed no significant change in secreted pro-BDNF at 4 and 24 h (Fig. 2a, d). No prepro-BDNF was detected in the extracellular media.

### ApoE2 increased, while ApoE4 decreased the expression of intracellular prepro-BDNF in astrocytes

**Fig. 3:**
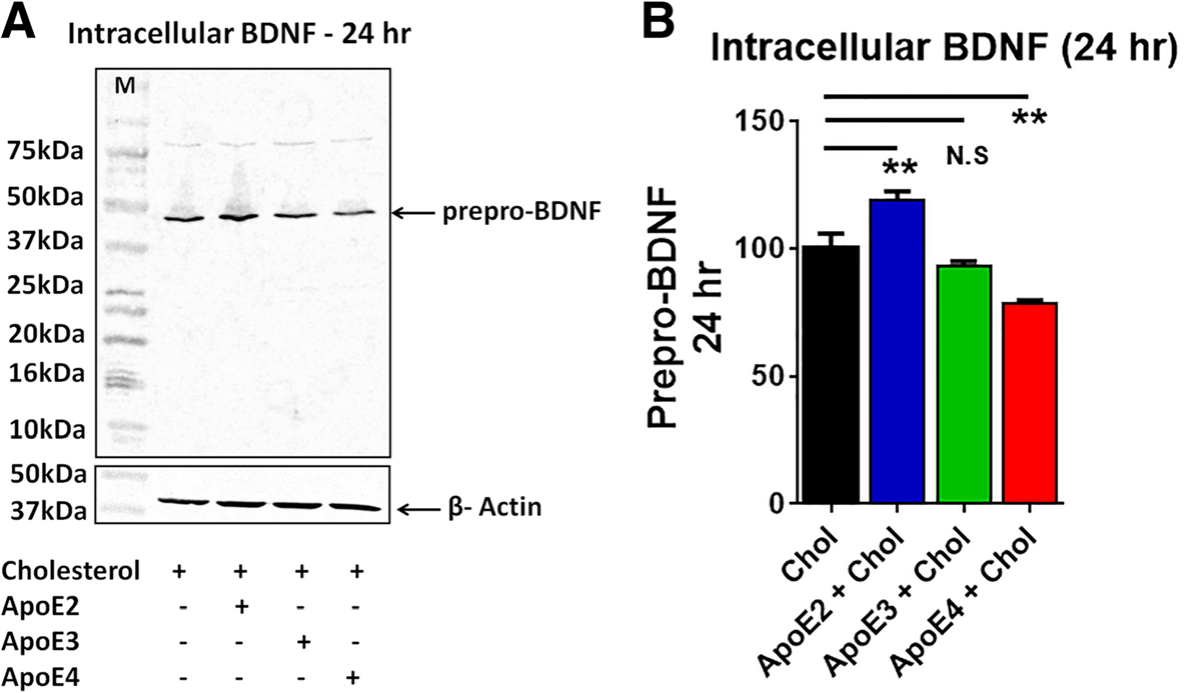
ApoE4 inhibits the expression of intracellular prepro-BDNF. Primary human astrocytes were treated with cholesterol (Chol; 100 μM), with or without ApoE2 (20 nM) or ApoE3 (20 nM) or ApoE4 (20 nM) for 24 h. **a** Immunoblot showing expression levels of BDNF in the cell lysate after 24 h. ‘M’ indicates molecular weight marker. **b** ApoE2 + cholesterol induces while ApoE4 + cholesterol inhibits BDNF synthesis. Data are represented as mean ± SE of three independent experiments (*p < .05; **p < .05; ***p < .005; one-way ANOVA, post hoc Newman-Keuls multiple comparison test)

We have shown earlier that ApoE4 suppress the expression of BDNF mRNA [9]. To determine if the ApoE isoforms differentially regulate the intracellular protein expression of BDNF, we measured the intracellular protein levels of BDNF from human astrocytes treated with cholesterol, ApoE2 + cholesterol, ApoE2 + cholesterol and ApoE4 + cholesterol for 4 and 24 h. At 4 h the amount of intracellular prepro-BDNF showed no significant difference in cells treated with different ApoE isoforms. After 24 h, the ApoE2 + cholesterol treated cells showed a 19.04 ± 6.68% increase, ApoE3 + cholesterol treated cells showed no significant change while ApoE4 + cholesterol treated cells showed a 21.61 ± 5.89% decrease in intracellular prepro-BDNF (ANOVA: F _(3,8)_ = 23.2; *P* = 0.0003) (Fig. 3a, b). Mature-BDNF and pro-BDNF were not detected in these fractions.

## Discussion

Brain-derived neurotrophic factor (BDNF) is involved in multiple aspects of synaptogenesis, from the formation to the functional maturation of synapses. BDNF influences the morphological complexity of axons and dendrites [15–17], increases synapse number [18–20], modulate synaptic maturation and controls ultrastructural composition of synapses [21, 22]. BDNF is critical for synaptic plasticity and memory processing in adult brain and is also essential to promote short and long term memory [23–26]. BDNF is synthesized as a precursor (pro-BDNF) encompassing two domains, the prodomain and the mature BDNF domain [27]. The pro-BDNF is cleaved by prohormone convertases such as furin and PC1/3 intracellularly or plasminogen/plasmin and MMPs extracellularly [28–30] to release the mature form [31].

Our data show that ApoE2 induces the astrocytes to secrete more m-BDNF than does ApoE3, while ApoE4 produces a negligible amount. We also found that the secreted pro-BDNF level was higher in ApoE3-treated astrocytes than in ApoE2-treated astrocytes. Our findings thus suggest that ApoE2 is critically involved in cleavage of pro-BDNF to m-BDNF. The m-BDNF binds to Trk-B receptors leading to cell survival and differentiation, whereas pro-BDNF binds to p75^NTR^ leading to apoptosis [32]. Infusion of exogenous pro-BDNF promotes synapse elimination, whereas m-BDNF infusion substantially delays synapse elimination [33]. Exogenous m-BDNF induces LTP and promotes long-term memory (LTM). ApoE4-transgenic mice have impaired long-term spatial memory with reduced dendritic spine density compared to ApoE3 or ApoE2 mice [34]. Persistent LTMs are associated with increased BDNF protein levels [26, 35]. Here we found that the total intracellular BDNF protein is lower in ApoE4-treated cells, higher in ApoE2-treated cells and remains unchanged in ApoE3-treated cells. We have demonstrated earlier, that ApoE3 induces and ApoE4 suppresses BDNF transcription, epigenetically by inducing nuclear translocation and association of HDAC6 to BDNF promoter IV [9]. Thus, ApoE4 is not only blocking the secretion and conversion of pro-BDNF to m-BDNF, but also preventing its synthesis via HDAC. Together our data indicate that ApoE regulates BDNF transcription, maturation and secretion in an isoform dependent manner. ApoE2 and ApoE3 may induce transcription, while ApoE4 inhibits transcription of BDNF via LRP1-HDAC pathway [9]. ApoE2 has very low binding affinity to LDLR (1–2% that of ApoE3; [36, 37]) and lower affinity than ApoE3 for LRP1 (40% of ApoE3 or ApoE4; [38, 39]). ApoE induces maturation and secretion of m-BDNF in an ApoE2 > ApoE3 > ApoE4 rank order. The differential maturation and secretion of BDNF could be regulated by the pro-BDNF cleaving enzymes viz. furin and plasmin in an ApoE-isoform dependent manner [40]. These findings correlate with the previous findings that BDNF expression and maturation is impaired by ApoE4 which may play an important role in the ApoE4 mediated deficit in long-term memory.

ApoE4*,* the greatest genetic risk factor for sporadic Alzheimer’s disease (AD), increases risk ~3- and 15-fold with a single or double allele [2, 41, 42] compared to ApoE3, whereas ApoE2 decreases AD risk ~2-fold per allele [43]. In EFAD transgenic mice generated by crossing 5xFAD mice and h-*APOE*-TR mice BDNF levels are decreased in the following pattern: E2FAD > E3FAD > 5xFAD/*APOE*-KO > E4FAD [44]. We found that BDNF protein levels are lower in human AD hippocampus, confirming the previous report that precursor form of BDNF and m-BDNF are decreased in the pre-clinical stages of Alzheimer’s disease [12].

Considering the common role of ApoE and BDNF in synaptogenesis, neuroprotection, synaptic plasticity and learning and memory, our results indicate a mechanistic link between ApoE isoforms and BDNF in maintaining synaptic plasticity. ApoE4 impairs the synthesis and secretion of BDNF which may cause synaptic loss and neuronal integrity [45, 46], reduced exitatory synaptic transmission and dendritic arborization [47] and loss of cognition and memory [44, 48, 49], leading to increased risk of AD. ApoE2 and ApoE3 induce BDNF expression and secretion and further downstream signaling pathways which may prevent the synaptic loss in AD [50]. ApoE2 overexpression ameliorates spine density loss in both Tg2576 and PDAPP mice [51]. ApoE3, but not ApoE4, protectected against neuronal damage in age-dependent neurodegeneration [52] and Aβ-oligomer-mediated synaptic loss through PKCε [8]. PKCε is another synaptogenic factor which shows a deficit in human AD hippocampus [53] and humanized ApoE4 transgenic mice [8]. PKCε activators (bryostatin 1, DCP-LA) have been shown to induce synaptogenesis [54] and prevent synaptic loss in aged and AD mice by inducing BDNF expression [55–57].

## Conclusion

The findings presented here, taken together, shows that ApoE isoforms differentially regulate BDNF expression and secretion and therefore, provide additional mechanistic insight as to how ApoE2 and ApoE3 may offer neuroprotection through BDNF, and how ApoE4 may cause neurodegeneration by inhibiting BDNF. It will be of future interest to clinically test how such defects in BDNF and neurodegeneration may be prevented by therapeutic use of PKCε activators [58].

## Abbreviations

AD: Alzheimer’s disease
ApoE: Apolipoprotein E
BDNF: Brain derived neurotrophic factor
